# Effects of Bariatric Surgeries on Male and Female Fertility: A Systematic Review

**Published:** 2020

**Authors:** Luz Cilis Moxthe, Rachel Sauls, Michelle Ruiz, Marilyn Stern, John Gonzalvo, Heewon L. Gray

**Affiliations:** 1- Department of Psychology, College of Arts and Sciences, University of South Florida, Tampa, Florida, USA; 2- Department of Child and Family Studies, College of Behavioral and Community Sciences, University of South Florida, Tampa, Florida, USA; 3- Advent Health Medical Group, Bariatrics at Tampa, Tampa, Florida, USA; 4- College of Public Health, University of South Florida, Tampa, Florida, USA

**Keywords:** Bariatric surgery, Fertility, Infertility, Obesity, Reproductive health, Systematic review

## Abstract

**Background:**

Morbid obesity has been known to decrease fertility in both men and women. This review aimed to examine current evidence of the effects of bariatric surgeries on fertility parameters including sex hormones in both men and women, seminal outcomes in men, menstrual cycle, PCOS symptoms, and pregnancy in women, and sexual function in both men and women.

**Methods:**

Three databases (PubMed, Web of Science, and Academic Search Premier) were used with key terms of bariatric surgery, bariatric surgical procedures, infertility, reproductive health, pregnancy, and fertility. Studies with male and/or female patients were included. Study types included retrospective chart reviews, observational, qualitative, cross-sectional, cohort, and longitudinal studies published in January 2008–June 2018. The search was performed on June 21–26, 2018. Quality assessment and data synthesis were conducted.

**Results:**

A total of 18 articles were included in the final review. Seven studies included only men, ten included only women, and one included both men and women. Bariatric surgery significantly improved hormonal balance and sexual functions in both males and females, sperm count in males, and pregnancy in females. The strongest evidence was found on bariatric surgery’s effects on sex hormones. No study with males asked whether the participants actually conceived a child with their partners after the bariatric surgery. Most weaknesses in all articles reviewed were lack of discussion on confounding variables and many did not differentiate surgical types.

**Conclusion:**

Bariatric surgery most effectively improved sex hormones. Further research is needed on direct pregnancy outcomes for both men and women.

## Introduction

Morbid obesity, defined as a BMI of 40 *kg/m*^2^ or more, has been known to decrease fertility in both men and women (1, 2). Obesity affects reproductive features and processes by disrupting normal hormone levels. For example, women with morbid obesity have higher levels of leptin produced from adipocytes, which can disrupt the hormonal balance in women, causing a change in menstrual cycle and fertility outcomes (3). Obesity can also lead to anovulation, exacerbating the symptoms of polycystic ovary syndrome (PCOS) (4). For men, obesity causes a reduction of sperm production and an increase in slow swimming that reduces the possibility of male fertility. For both men and women, disrupted levels of reproductive hormones, such as sex hormone binding globulin (SHBG), follicle-stimulating hormone (FSH), and testosterone have also been linked to obesity, resulting in infertility (5).

Bariatric surgery is the most effective method to treat morbid obesity and can be categorized as either restrictive or malabsorptive (6). Restrictive surgery involves restricting food intake, while malabsorptive surgery limits the amount of nutrients and calories that can be digested (7). The most commonly used procedures worldwide are laparoscopic adjustable gastric band, sleeve gastrectomy, and gastric bypass. In laparoscopic adjustable gastric band surgery, an inflatable band is placed around the stomach, allowing for a small amount of food to create the feeling of being full (8, 9). Sleeve gastrectomy involves removing part of the stomach, restricting the amount of food eaten (10). Gastric bypass, or Roux-en-Y gastric bypass, involves both restrictive and malabsorptive methods (11). The stomach is made smaller and the small intestine is rerouted to where food bypasses the majority of the stomach and upper intestine to reduce the amount of calories absorbed (4). Previous research studies suggest that these various bariatric surgeries can effectively improve fertility for patients with obesity. However, a systematic review of the current evidence on the efficacy of bariatric surgeries on improving fertility among both men and women is not available.

Two systematic reviews on bariatric surgery and maternal and neonatal outcomes in pregnant women (12, 13) found that bariatric surgery reduced the risk of adverse outcomes in women and infants. Similarly, a study compared bariatric surgery to non-surgical weight loss interventions and found that the rapid weight loss from bariatric surgery increased fertility immediately in comparison to gradual weight loss over time, but only in women (14). Another systematic review and meta-analysis found that non-surgical weight loss interventions improved fertility in both men and women who are overweight or obese (15). However, there were only two studies that included men, and the authors reported that neither of these studies provided adequate details of the intervention, concluding that there was little evidence to determine the effectiveness of weight loss on fertility outcomes in men.

In summary, bariatric surgeries are known to be most effective in reducing substantial weight among people with morbid obesity. There have been several reviews on the impact of bariatric surgeries on pregnancy, fertility, and infant outcomes in women (12, 16–20). Non-surgical weight-loss interventions seemed to improve fertility in women and men (15), yet there has been no systematic review on the impact of bariatric surgeries on fertility parameters in both men and women. The purpose of this review was to systematically examine current evidence of the effects of bariatric surgeries on fertility outcomes in both men and women. Outcome of interest was evaluation of infertility/fertility, polycystic ovary syndrome (PCOS), sex hormones, pregnancy, and semen parameters.

## Methods

### Search strategy:

This review was conducted to identify published, peer-reviewed journal articles that assessed fertility in men and women who have undergone bariatric surgery. The search was performed on three databases including PubMed, Web of Science, and Academic Search Premier, using PRISMA procedures. Key terms were developed and searched in each of the three databases including bariatric surgery, bariatric surgical procedures, infertility, reproductive health, pregnancy, and fertility. All articles published from January 2008 to June 2018 were included. The keyword search was completed by the team on June 21, 2018. Search strategies included using a combination of the key terms, (((Infertility [MeSH] OR Infertility OR Reproductive health [MeSH] OR Pregnancy [MeSH] OR Fertility) AND “last 10 years” [PDat] AND English [lang])) AND ((Bariatric surgery [MeSH] OR “Bariatric surgery” OR Bariatrics [MeSH] OR “Bariatric Surgical Procedures”) AND “last 10 years” [PDat] AND English [lang]). Three researchers (LCM, MR, RS) were independently involved in the search process. The protocol was registered on the International Prospective Register of Systematic Reviews system PROSPERO (CRD42018096965).

### Inclusion and exclusion criteria:

Studies with bariatric surgery and fertility outcomes for both male and female were included. Fertility outcomes included self-reports of infertility, PCOS, sex hormones, menstruation, pregnancy, semen parameters, and any other indicators used to measure fertility. Peer reviewed articles were reviewed and types of study designs included were retrospective chart reviews, observational studies, qualitative studies, cross-sectional, cohort, and longitudinal studies. The following exclusion criteria were applied; bariatric surgery occurred during pregnancy, participants did not undergo any bariatric surgery, and sexual behavior outcomes were obtained without testing fertility or pregnancy. Conference papers, case studies, newsletters, commentaries, opinion articles, editorials, thesis or dissertations without peer-review were excluded.

### Data extraction:

Three reviewers (LCM, MR, RS) independently extracted the following data from each study: gender, age, surgery type, body composition changes, weight evaluation timeframe, statistical tests, p-values, fertility and pregnancy outcomes. Fertility outcomes included any information about fertility or infertility improvements, PCOS, sex hormones, semen parameters, pregnancies per participant, pregnancy complications, and other long term outcomes. To ensure accurate data extraction of each article, any discrepancies were discussed amongst the research team until complete agreement was reached. Two reviewers (MS and HLG) reviewed and confirmed the results of the data extraction.

### Quality assessment:

The articles used for the literature review underwent a quality assessment through the National Institutes of Health (NIH) guidelines on Systematic Evidence Reviews (21). The quality assessment presented 14 questions regarding the rigor of each study and potential biases. Three raters (LCM, MR, RS) independently reported each item with either a “Yes”, “No”, “Not applicable”, or “Not Reported”. To complete an inter-reliability assessment of discrepancies in the quality assessment, the raters were given articles that overlapped to compare results. The final quality of each article was classified as “Good”, “Fair”, or “Poor” based on the overall quality and risks for potential biases. Discrepancies between the raters were calculated by the percent agreement and discussed until perfect agreement was reached on a finalized quality assessment for each article.

### Data synthesis:

Study characteristics were organized in a table with study design, gender included, initial weight/BMI of the participants, weight reduction after the surgery, and fertility/pregnancy outcome measures. Using the data from the data extraction process, main study outcomes and effect sizes were separately synthesized into a table. The data were synthesized independently by three reviewers (LCM, MR, RS) using information derived on types of bariatric surgery, detailed fertility outcome measures, statistical tests, significance, and effect sizes. Any discrepancies were discussed until 100% agreement to ensure accurate data synthesis. Due to heterogeneity of surgery type and outcomes measured in each study, a quantitative meta-analysis was not performed.

## Results

### Study selection:

**Figure 1.**
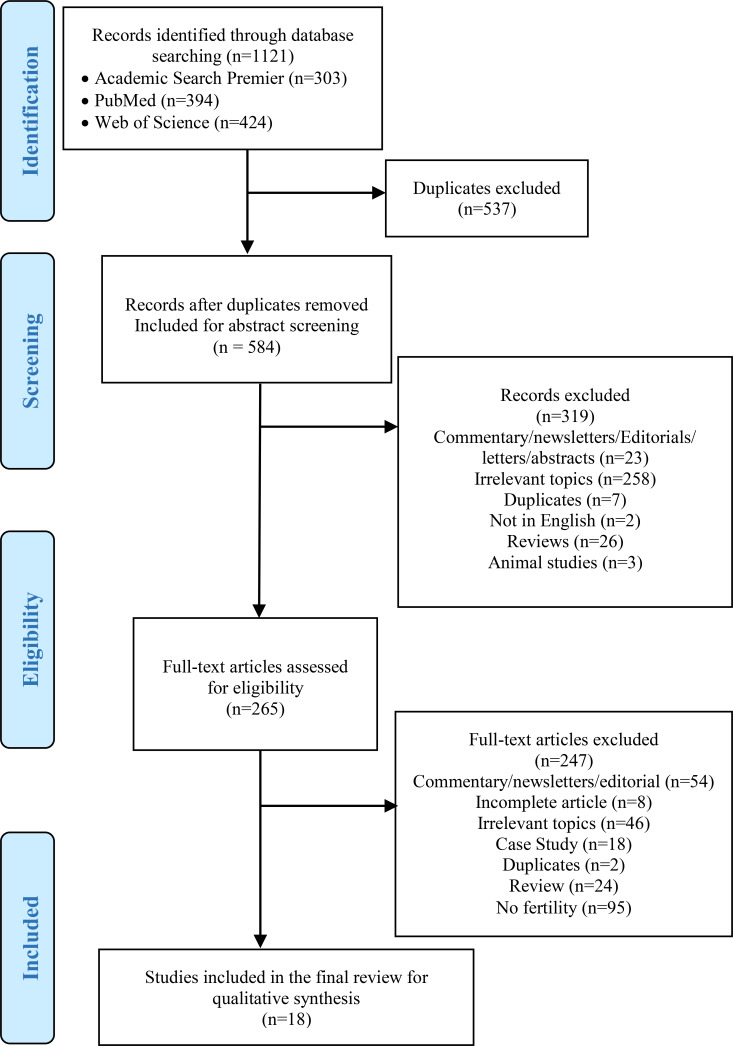
PRISMA 2009 Flow Diagram

Figure 1 illustrates the study selection process. The initial database search identified 1,121 articles, 303 from Academic Search Premier, 394 from PubMed, and 424 from Web of Science. Afterward, 537 duplicate articles were identified and removed, resulting in 584 articles for further investigation. The abstracts for the 584 articles were subsequently reviewed and 319 articles were excluded. The exclusion of the 319 articles were for the following reasons: 23 were found to be commentary/editorials/or abstracts, 258 were on irrelevant topics, 7 were duplicates, 2 were non-English publications, 26 were reviews, and 3 were animal studies. As a result, 265 articles were included from the abstract screening process. A full-text article review was further completed, and 247 articles were excluded for the following reasons: 54 articles were commentary/newsletter/editorials, 8 incomplete articles, 46 irrelevant topics, 18 case studies, 2 duplicates, 24 reviews, and 95 studies did not mention fertility. A total of 18 articles assessing fertility after bariatric surgery were included in the final review. Seven studies included only men, ten studies included only women, and one article reported on both male and female patients.

### Study characteristics:

Table 1 summarizes the study characteristics that were reviewed in this study. Most studies used prospective cohort or retrospective chart review. One study used a randomized controlled trial. The age range included was between 18 and 49, and the mean BMI before surgery was over 40 for all studies, ranging from 41 to 71 *kg/m*^2^. All studies reported weight or BMI changes after surgical procedures. Outcome measures after surgery were conducted mostly in 6 and 12 months, up to 24 months in prospective cohort studies or backtracked up to 5 years in retrospective studies. All seven studies including only male patients assessed sex hormones as outcomes. Sex hormone binding protein, semen or sperm parameters, sexual quality of life, and international index of erectile function were other outcome measures in male patients. Four studies assessed seminal outcomes (22–25). One study assessed sexual quality of life (26) and two studies assessed erectile function (23, 25). Studies with female patients assessed menstrual regularity or dysfunction, PCOS, self-reported infertility and pregnancy history, hirsutism, sex hormones, and sexual functions as outcomes. Seven out of eleven studies that included female patients examined conception/pregnancy as an outcome, while nostudy with male patients tracked whether they actually had successful conception or pregnancy with their partners after the surgery. Variables and definitions included in the selected studies in the review are summarized in table 2.

**Table 1.** Study characteristics of the studies examining the effects of bariatric surgeries on fertility outcomes

**Study citation**	**Study design**	**n.**	**Sex**	**Age**	**Type of surgery**	**Initial weight**	**Weight evaluation timeframe**	**Weight changes after surgery**	**Fertility outcomes**
Facchiano E, et al. (2013)	Prospective cohort	20	M	27–47	LGBP/LGB/LBPD	BMI 40.5	6 months	Mean BMI 34.8	Sex hormones, sex hormone binding protein
El Bardisi H, et al. (2016)	Prospective cohort	50	M	29–44	LSG	BMI 71.4	12 months	Median BMI 22.8	Sex hormones, semen normalized, sperm detection with azoospermia, and sperm count
Hammoud A, et al. (2009)	Prospective cohort	64	M	Mean 49	RYGB	BMI 46.2	2 years	Mean BMI −16.6	Sex hormones and sexual quality of life
Legro RS, et al. (2015)	Prospective cohort	6	M	18–40	RYGB	BMI 49	1, 3, 6,12 and 24 months	BMI −6, −11, −15, −18, −19 at follow up points	Serum sex hormones and sex hormone binding protein, semen volume, concentration, and motility, and erectile function
Luconi M, et al. (2013)	Prospective cohort	24	M	31.2–46.7	RYGB/AGB/BPD	BMI 43.9; 139.2 *kg*	6 and 12 months	6M=105 *kg*, 12M=104 *kg*	Sex hormones and sex hormone binding protein
Samavat J, et al. (2018)	Two-armed prospective cohort	31	M	Not Specified	LRYGB	Not Specified	6 months	Not Specified	Sex hormones, sperm motility, sperm number, semen volume and concentration
Reis LO, et al. (2012)	Randomized controlled trial	20	M	Mean 39.3	RYGB	Weight 168.6 *kg*; BMI 55.7	T1=4 months nonsurgical intervention, T2=20 months after surgery	Mean BMI −12.6 at T1 & −24.7 at T2.	International Index of Erectile Function (IIEF-5) questionnaire, sex hormones, sperm volume, motility, normal morphology
Edison E, et al. (2016)	Retrospective	15,222	F	18–45	RYGB/AGB/LSG/G astric Balloon/BPD Duodenal Switch	BMI 48.2	12 months	Mean BMI 37.3	Menstrual dysfunction, PCOS
Goldman RH, et al. (2016)	Pre- and post-test between surgery and control group	219	F	18–45	RYGB/AGB	BMI >35	18 months	BMI −14.71±6.35 in the RYGB group and −9.17±6.16 in the AGB group	Menstrual regularity, self-reported infertility/pregnancy history (term birth, miscarriage, live birth, and birth weight)
Jamal M, et al. (2012)	Cross-sectional	20	F	22–42	RYGB	BMI 52.8±9.08	3–5 years	Mean BMI 34.3±5.7	Menstruation regularity, hirsutism, PCOS, and conception
Khazraei H, et al. (2017)	Retrospective chart review	221	F	Mean 36	LSG	Mean BMI 44; 116.31 *kg*	12 months	Mean −40.09 *kg*	Pregnancy, menstruation, hirsutism
Kjaer MM, et al. (2017)	Prospective cohort	31	F	Mean 34 (22–49)	RYGB	BMI 44.1±5.8	3, 6 and 12 months	BMI 35.5±5.2, 32.4±4.9, 30.3±5.8 at follow up points	PCOS, irregular menstrual periods, and sex hormones.
Legro RS, et al. (2012)	Prospective cohort	29	F	Mean 34.5±4.3	RYGB	BMI 49±7; 132 *kg*±17	1, 3, 6, 12, and up to 24 months	−15, −29, −40, −50, −51 *kg* at follow up points	Conception, menstruation, sexual function, SHBG, sex hormones
Luyssen J, et al. (2018)	Prospective cohort	71	F	18–43	LRYGB/LSG	BMI 42.0	6 and12 months	−34.9±7.2 *kg*, −43±9.6 *kg*	Menstrual frequency, pattern, cycle and duration, intimate relationship, frequency of sex, and sexual satisfaction.
Musella M, et al. (2011)	Retrospective chart review	23	F	Mean 31±4.8 (22–39)	Intragastric Balloon	BMI 41±2.7	At least 1 year follow up	Mean BMI −7.5±1.1	Pregnancy, infertility defined as inability to carry pregnancy to live birth after one year of regular unprotected sex
Musella M, et al. (2012)	Case control retrospective chart review	110	F	Pregnant: 29.3±3.9, Non-pregnant: 28.6±3.2	Intragastric Balloon/AGB/LSG/RY GB	BMI Pregnant: 43.9±4.1, Non-pregnant: 45.1±3.7	2.5 years	BMI Pregnant: 34.2±2.4, after Non-pregnant to make it consistent 41.5±2.8	Pregnancy and pregnancy complications.
Nilsson-Condori E, et al. (2018)	Prospective cohort	48	F	18–35	RYGB	BMI 40.9	12 months	BMI 25.4	Sex hormones
Laurino Neto RM, et al. (2012)	Retrospective chart review	140	M/F	Mean 41.4 (19–62)	RYGB	BMI 52.5	1, 3, 6 and 12 months	BMI 33.7 at last follow up	Amenorrhea, irregular menstrual cycles, inability to become pregnant after 6 months

M=Male; F=Female; LGBP=Laparoscopic Gastric Bypass; LGB=Laparoscopic Banding; LBPD=Laparoscopic Biliopancreatic Diversions; LSG=Laparoscopic Sleeve Gastrectomy; RYGB=Roux-en-Y Gastric Bypass; AGB=Adjustable Gastric Band; BPD=Biliopancreatic Diversion; LRYGB=Laparoscopic Roux-en-y Gastric Bypass; LSG=Laparoscopic Sleeve Gastrectomy; BMI=Body Mass Index; PCOS=Polycystic Ovary Syndrome; SHGB=Sex Hormone-Binding Globulin

**Table 2.** Results of the Quality Assessment

**Items**	**Edison E, et al. (2016)**	**El Bardisi H, et al. (2016)**	**Facchiano E, et al. (2013)**	**Goldman RH, et al. (2016)**	**Hammoud A, et al. (2009)**	**Jamal M, et al. (2012)**	**Khazraei H, et al. (2017)**	**Laurino Neto RM, et al. (2012)**	**Legro RS, et al. (2012)**	**Legro RS, et al. (2015)**	**Luconi M, et al. (2013)**	**Luyssen J, et al. (2018)**	**Musella M, et al. (2011)**	**Nilsson-Condori E, et al. (2018)**	**Reis LO, et al. (2012)**	**Samavat J, et al. (2018)**	**Kjaer MM, et al(2017)**	**Musella M, et al. (2012)**
1. Was the research question or objective in this paper clearly stated?	O	O	O	O	O	O	O	O	O	O	O	O	O	O	O	O	O	O
2. Was the study population clearly specified and defined?	O	O	O	O	O	O	O	O	O	O	O	O	O	O	O	O	O	O
3. Was the participation rate of eligible persons at least 50%?	O	O	NR	X	X	X	NR	NR	O	O	O	O	O	NR	X	O	NR	O
4. Were all the subjects selected or recruited from the same or similar populations (including the same time period)? Were inclusion and exclusion criteria for being in the study pre-specified and applied uniformly to all participants?	O	O	O	O	X	O	O	O	O	O	O	X	O	NR	O	O	O	O
5. Was a sample size justification, power description, or variance and effect estimates provided?	X	X	O	X	X	O	X	X	O	O	X	X	X	X	X	X	X	X
6. For the analyses in this paper, were the exposure(s) of interest measured prior to the outcome(s) being measured?	O	O	O	O	O	O	O	O	O	O	O	O	O	O	O	O	O	O
7. Was the timeframe sufficient so that one could reasonably expect to see an association between exposure and outcome if it existed?	O	O	X	O	O	O	O	O	O	O	O	O	O	O	O	X	O	O
8. For exposures that can vary in amount or level, did the study examine different levels of the exposure as related to the out-come (e.g., categories of exposure, or exposure measured as continuous variable)?	O	X	O	O	X	X	X	X	X	X	O	X	X	X	X	X	X	O
9. Were the exposure measures (independent variables) clearly defined, valid, reliable, and implemented consistently across all study participants?	O	O	O	O	O	O	O	O	O	O	O	O	O	O	O	O	O	O
10. Was the exposure(s) assessed more than once over time?	NA	NA	NA	NA	NA	NA	NA	NA	NA	NA	NA	NA	NA	NA	NA	NA	NA	NA
11. Were the outcome measures (dependent variables) clearly defined, valid, reliable, and implemented consistently across all study participants?	X	O	O	O	O	O	O	O	O	O	O	O	O	O	O	O	O	O
12. Were the outcome assessors blinded to the exposure status of participants?	NR	NR	NR	NR	NR	NR	NR	NR	NR	NR	NR	NR	NR	NR	NR	NR	NR	NR
13. Was loss to follow-up after baseline 20% or less?	O	O	O	O	X	O	NR	X	X	O	O	X	O	O	O	O	O	O
14. Were key potential confounding variables measured and adjusted statistically for their impact on the relationship between exposure(s) and outcome(s)?	No	No	O	O	No	No	O	No	No	No	O	No	No	O	O	No	No	O
Rating: (based on the guide rather than the total score)	Fair	Fair	Fair	Fair	Fair	Fair	Good	Fair	Fair	Fair	Good	Fair	Fair	Poor	Good	Fair	Good	Good

O=Yes; X=No; N/A=Not applied; NR=Not reported

### Quality assessment:

Table 3 presents the results from the quality assessment. An average interrater reliability was 86% in the original quality assessment conducted by the three raters in assessing the 18 articles. After discussing the quality assessment of the criteria for each question, the three raters were able to agree on their results to yield an overall 100% agreement for the quality assessment. The overall ratings of the final group of articles reviewed ranked 5 as good, 12 as fair and 1 as poor. Most studies had a clear explanation of aim, population size, and participation rate. However, two studies did not clearly discuss the independent variable as bariatric surgery with regard to the different types of bariatric surgery used. As a result, not every study mentioned how outcomes were altered from different types of bariatric surgeries used. Five of the eighteen articles reported a loss of over 20% of followed up participants after the initial baseline assessment. Eleven out of eighteen did not report confounding variables in their results that could have an impact on the data and statistics from this research. Two studies did not report accurately the amount of time elapsed between the bariatric surgery procedure and the first follow up, thereby reducing the ability to evaluate degree of improvements in fertility. Most of the articles ranked as fair because they lacked a discussion of confounding variables and failed to differentiate between bariatric surgery types.

**Table 3.** Significant effects of bariatric surgeries on fertility outcomes in male and female patients

**Citation**	**Sex**	**Surgery type**	**Outcomes**		**Effects and significance**			
Facchiano E, et al. (2013)								
	M	LGB/LGBP/LBPD	Collected at baseline and 6 months after surgery					

			Total Testosterone (*nM*)		↑ from 8.1 to 13.2, p<0.0001			

			Total Estradiol (*pmol/l*)		↓ from 149.5 to 112, p=0.002			

			FSH (*mlU/ml*)		↑ from 3.28 to 4.17, p<0.0001			

			LH (*mlU/ml*)		↑ from 2.7 to 3.62, p=0.048			

			SHBG (*nM*)		↑ from 19.0 to 39.4, p<0.0001			

			Total Testosterone and Age association		Men under 35 saw more of an increase p=.043 than men 35 years or older			

			Free Testosterone and Age association		Men under 35 saw more of an increase p=.005 than men 35 years or older			

El Bardisi H, et al. (2016)								
	M	LSG	Collected at baseline and 12 months after surgery					

			Testosterone (*nmol/l*)		↑ from 16.4 to 22.4, p<.001			

			Compared by sperm count groups		Azoospermia (0.00 *M/ml*)	Oligospermia (<15 *M/ml*)	Normal (>15 *M/ml*)	

			Testosterone (*nmol/l*)		↑ from 13.8 to 18.8, p<0.001	↑ from 17.4 to 22.4, p<0.001	↑ from 13.7 to 22.4, p<0.001	

			Sperm count (*M/ml*)		↑ from 0.00 to 0.06, p<0.05	↑ from 1.95 to 7.1, p<0.05	NS	

Hammoud A, et al. (2009)								
	M	RYGB	Outcome changes compared at 2 years		Surgery	Control	p-value	

			Total Testosterone (*ng/dl*)		↑ 310.8±47.6	↑ 14.2±15.3	p<0.001	

			Free Testosterone (*pg/ml*)		↑ 45.2±5.1	↑ 0.4±3.0	p=0.047	

			Estradiol (*pg/ml*)		↓ −8.1±2.4	↑ 1.6±1.4	p=0.006	

			SHBG (*nmol/l*)		↑ 21.6±2.8	↑ 2.3±0.8	p<0.001	

			C-reactive protein (*mg/dl*)		↓ −0.5±0.1	↓ −0.0±0.05	p<0.001	

			Within group changes at 2 years		Surgery	Control	p-value	

			Avoid sexual encounters		−1.8±0.3	−0.0±0.2	p<0.001	

			Difficulty with sexual Performance		−2.3±0.3	−0.1±0.2	p<0.001	

			Have little sexual desire		−1.9±0.2	0.05±0.2	p<0.001	

			Do not enjoy sex		−1.7±0.3	−0.05±0.2	p<0.001	

			Total score of dissatisfaction		−7.5±1.2	−0.1±0.6	p<0.001	

Legro RS, et al. (2015)								
	M	RYGB	Follow up after surgery		1 month ^a^	3 month ^a^	6 month ^a^	12 month ^a^

			Serum testosterone (*nmol/l*)		↑ 5 (2, 8), p<0.01	NS	↑ 6 (2, 11), p= 0.01	NS

			Serum SHBG (*nmol/l*)		↑ 24 (15, 32), p<0.01	↑ 17 (5, 29), p=0.01	↑ 21 (6, 37), p=0.01	↑ 25 (5, 46), p=0.02

			Free androgen index		↓ −12 (−21, −2), p= 0.02	NS	NS	NS

			Urinary total testosterone (*ng/mg cr*)		NS	↑, p<0.001	↑, p<0.001	↑, p<0.001

			Urinary creatinine (*mg/ml*)		↑, p<0.001	↑, p<0.001	NS	NS

Luconi M, et al. (2013)								
	M	RYGB/AGB/BPD	Relation of BMI and TT and SHBG		Baseline BMI was a significant predictor of variation in both TT (age-adjusted r=0.62, p=.009) and SHBG (age-adjusted r=0.54, p=.025) at 12-month follow-up			

			Correlation of BMI loss (DBMI) with extra TT gain		Significant correlation of BMI loss (DBMI) with extra TT gain (r=0.62; p=.001) at 6 months and maintained at 12 months (r=0.49, p=.025)			

			Correlation of BMI loss (DBMI) with extra SHBG gain		Significant correlation of BMI loss (DBMI) with extra SHBG gain (r=0.46; p=.025) at 6 months and maintained at 12 months (r=0.53, p=.013)			

			Longitudinal outcomes		At 6 months		At 12 months	

			TT (*nM*)		↑ 14.8, p<0.001		↑ 13.90, p<0.001	

			E2 (*pM*)		↓ 115.5, p=0.001		↓ 129, p=0.01	

			TT/E2		↑ 94, p<0.001		↑ 87, p<0.001	

			FSH (*mlU/l*)		↑ 4.17, p<0.001		↑ 5.33, p=0.001	

			LH (*mlU/l*)		↑ 3.62, p=0.010		↑ 3.54, p=0.004	

			CFT (*nM*)		↑ .265, p=0.021		↑ .271, p=0.050	

			SHGB (*nM*)		↑ 40.0, p<0.001		↑ 38.5, p<0.001	

Samavat J, et al. (2018)								
	M	Not Specified	Mean change at 6 months		Surgery		Control	

			TT		↑ from 9.00±4.00 to 15.24±4.25, p<0.001		NS	

			E2 (*nM*)		↓ from 150.1±38.3 to 116.6±43.6, p=0.003		NS	

			T/E2 (*nM/pM*)		↑ from 0.064±0.029 to 0.150±0.079, p<0.001		NS	

			FSH (*mlU/l*)		↑ from 3.74±2.25 to 5.59±3.02, p<0.001		NS	

			LH (*mlU/l*)		↑ from 2.54±1.69 to 4.06±1.33, p<0.001		NS	

			SHGB (*nM*)		↑ from 20.0±8.8 to 39.0±16.7, p<0.001		NS	

			CFT (*nM*)		↑ from 0.228±0.094 to 0.297±0.074, p=0.002		NS	

			Seminal Outcomes at 6 months		Surgery		Control	

			Viability (%)		↑ from 68.6±13.4 to 79.5±10.3, p=0.029		NS	

			Sperm Volume (*ml*)		↑ from 2.2±1.3 to 2.8±1.4, p=0.044		NS	

			Correlations with BMI variations		Univariate analysis		Multivariate analysis	

			TT		p<0.001		p=0.001	

			Sperm morphology		p=0.019		p=0.025	

			Sperm number		p=0.002		p<0.001	

			Semen volume		p=0.003		p=0.015	

Reis LO, et al. (2012)								
	M	RYGB	Within-group at 24 months		Surgery		Control	

			IIEF-5		↑ from 19.7±6.6 to 23.0±2.3, p= 0.0469		NS	

			FSH (*mUI/ml*)		↑ from 4.0±3.4 to 7.4±7.5, p= 0.0025		NS	

			TT (*ng/ml*)		↑ from 3.4±1.3 to 7.0±0.8, p=0.0349		NS	

			Prolactin (*μUI/ml*)		↑ from 14.1±7.8 to 6.8±3.2, p< 0.001		NS	

			Between-group at 24 months		Surgery	Control	p-value	

			IIEF-5		23.0±2.3	17.3±6.7	p=0.0224	

			TT (*ng/ml*)		7.0±0.8	2.9±0.4	p=0.0043	

			Free Testotsterone (*pg/ml*)		12.7±2.5	8.4±1.7	p=0.0149	

Edison E, et al. (2016)								
	F	RYGB/AGB/LSG/Gastric Balloon/BPD/Duodenal Switch	Collected at baseline and 12 months after surgery					

			Menstrual dysfunction		12.4% ↓, p<.001			

			PCOS		Diagnosis 14.8% ↓, p<.001			

Goldman RH, et al. (2016)								
	F	RYGB/AGB	Menstrual cycle irregularity compared among different study groups (no surgery, RYGB, AGB)					

			Pre-(referent) *vs*. post-RYGB		OR 0.21 (0.07–0.61), p<0.05			

			RYGB (referent) *vs*. AGB		OR 0.33 (0.12–0.87), p<0.05			

			Post-AGB *vs*. no surgery (referent)		OR 0.23 (0.06–0.96), p<0.05			

Jamal, M., et al. (2012)								
	F	RYGB	Hirsutism		Resolved in 29% (n=14 to 10), p<0.005			

			Menstrual dysfunction		82% corrected (n=17 to 3), p<0.005			

			Pregnancy in infertile PCOS subjects		100% conception			

Khazraei H, et al. (2017)								
	F	LSG	Irregular menstruation		Women with infertility, 40% became regular (60% to 20%)			

			Hirsutism		Women with infertility, 10% cured (50% to 40%)			

			Pregnancy rate		46.67% (n=7/15) tried unsuccessfully to become pregnant became pregnant			

Kjaer MM, et al. (2017)								
	F	RYBG	Collected at baseline, 3 months, 6 months, and 12 months					

			PCOS		Cases with symptoms 87% ↓ (n=8 to 1) at 3 months post-operative			

			Menstruation Regularity		85% (11/13) became regular at 12 months in women with oligo-/amenorrhea			

			Hormones, all at p<0.05		0 to 3 months	3 to 6 months	6 to 12 months	0 to 12 months

			SHBG (*nmol/l*)		61.1±24.7 ↑	76.0±21.5 ↑	85.6±24.6 ↑	85.6±24.6 ↑

			Testosterone (*nmol/l*)		0.90±0.34 ↓	NS	NS	0.92±0.29 ↓

			Free testosterone (*nmol/l*)		0.015±0.008 ↓	0.011±0.005↓	NS	0.012±0.005 ↓

			Modified FG-score (mean)		NS	NS	4.1±5.2 ↓	NS

			Androstendione (*nmol/l*)		3.23±1.29 ↓	NS	NS	NS

			Dehydroepiandrosterone (*nmol/l*)		3060.3±1489.3↓	NS	NS	3262.5±1687.1↓

			LH/FSH ratio		0.98±0.72 ↑	NS	NS	NS

			Estrone (*pmol/l*)		NS	127.8±69.9 ↓	NS	104.0±59.8 ↓

			Estronesulfate (*pmol/l*)		NS	NS	NS	1581.6±1133.4↓

Legro RS, et al. (2012)								
	F	RYGB	Conception	Five women conceived after surgery, four of whom had prior pregnancies				

			Sexual function	21.2±9.6 at baseline vs. 27.1±7.4 at 12 months, p=0.02				

			SHBG	↑ immediately within 1 month of surgery (p<0.001)				

			Testosterone	↓ primarily in the 3-month postoperative period (p=0.002)				

			Estradiol	↑ only at month 6 (p=0.03)				

			Free androgen index	↓ within 1 month of surgery (p<0.001)				

			Menstrual cycle parameters	at 1 month	at 3 months	at 6 months	at 12 months	at 24 months

			Menstrual cycle length (d)	NS	NS	↓ −6.0 (−11.7, −0.3), p=0.04	NS	NS

			Follicular phase length (d)	NS	↓ −6.5 (−10.5, −2.4), p=0.002	↓ −8.2 (−12.3, −4.2), p<0.001	↓ −7.9 (−12.1, −3.7), p<0.001	↓ −8.9 (−13.9, −3.9), p<0.001

			Luteal phase length (d)	↓ 3.8 (0.4, 7.2), p=0.03	NS	NS	NS	NS

			Ovulatory cycles (%)	NS	NS	↓ 10.1 (0.2, 20.0), p=0.05	NS	NS

			Creatinine (*mg/ml*)	↑ 1.16 (0.87, 1.46), p<0.001	↓ 0.94 (0.64, 1.24) p<0.001	↓ 0.58 (0.27, 0.89), p<0.001	↓0.40(0.08, 0.72), p=0.02	NS

Luyssen J, et al. (2018)								
	F	LRYGB/LSG	Collected preoperatively, 6 months after, and 12 months after surgery					

			Menstrual frequency, pattern, cycle and duration, intimate relationship, frequency of sex, and sexual satisfaction					NS

Musella M, et al. (2011)								
	F	Intragastric Balloon	Pregnancy obtained through IVF		All four patients who previously underwent failed IVF obtained a pregnancy through IVF after surgery			

			Pregnancy obtained naturally		78.5% (n=11/14) unable to achieve a pregnancy did after surgery			

			Overall conception		83.3% (n=15/18) who were unsuccessful in becoming pregnant became pregnant			

Musella M, et al. (2012)								
	F	Intragastric Balloon/AGB/LSG/RYGB	Overall pregnancy		62.7% who could not conceive became pregnant after surgery			

			BMI in pregnant *vs*. non-pregnant group		34.2±2.4 pregnant group *vs*. 41.5±2.8 non-pregnant group, p=.001			

			% patients >5 BMI weight loss in pregnant vs. non-pregnant group		91% (n=63/69) pregnant group *vs*. 34% (n=14/41) non-pregnant group, p=0.001			

Nilsson-Condori E, et al. (2018)								
	F	RYGB	Hormones, all at p<0.05	baseline and at operation	baseline and 6 months		baseline and 12 months	

			AMH (*pmol/l*)	↑ 35.0 (4.1–160.0)	↓ 19.5 (2.0–83.0)		↓ 18.0 (2.0–84.0)	

			Testosterone (*nmol/l*)	NS	↓ 1.0 (0.2–2.3)		↓ 0.9 (0.2–2.3)	

			SHBG (*nmol/l*)	↑ 39.5 (10.0–199.0)	↑ 67.0 (1.8–157.0)		↑ 73.0 (21.0–270.0)	

			Free androgen index (FAI)	NS	↓ 1.5 (0.1–61.1)		↓ 1.2 (0.1–4.0)	

			Estradiol (*pmol/l*)	↑ 312.5 (100.0–2378.0)	↑ 314.0 (20.0–15780.0)		↑ 306.0 (20.0–3719.0)	

			Androstenedione (*nmol/l*)	NS	↓ 4.2 (1.8–14.5)		↓ 3.8 (1.3–9.3)	

			DHEAS (*μmol/l*)	↑ 6.0 (1.9–13.0)	↓ 4.3 (1.2–9.6)		↓ 4.5 (1.5–12.0)	

Laurino Neto RM, et al. (2012)								
	M/F	RYGB	Collected preoperatively, 1, 3, 6, and 12 months after operation and yearly thereafter					

			Amenorrhea, irregular menstrual cycles, inability to become pregnant					NS

M=Male; F=Female; NS=Not significant; FSH=Folliculer-Stimulating Hormone; LH=Luteinizing Hormone; SHBG=Sex Hormone Binding Globulin; IIEF=International Index of Erectile Function; a=mean (95% Confidence Interval); PRL=prolactin; TT=total testosterone; PCOS= polycystic ovary syndrome, AGB=adjustable gastric band, OR=odds ratio

### Effects of bariatric surgery on fertility:

The results of the data synthesis are summarized in table 4. Only significant outcomes are presented in the table and the studies are organized by participants’ sex (Studies with male patients, female patients, and both sexes). The three major outcome categories examined in male patients were sex hormones, seminal outcomes such as sperm counts, and sexual function and satisfaction. For female patients, fertility outcomes included five major categories of sex hormones, menstrual regularity or dysfunction, PCOS, conception/pregnancy, and sexual function and satisfaction.

**Table 4.** Variables and definitions included in the selected studies in the review

**Variable**	**Definition**
Total Testosterone (*nM*)	The amount of the male hormone, testosterone, in the blood
Total Estradiol (*pmol/l*)	Female hormone, produced primarily in the ovary. The amount of estrogen produced depends on the phase of the menstrual cycle
FSH (*mlU/ml*)	Follicle-stimulating hormone gonadotropin, a glycoprotein polypeptide hormone. FSH is synthesized and secreted by the gonadotropic cells of the anterior pituitary gland, and regulates the development, growth, pubertal maturation, and reproductive processes of the body
LH (*mlU/ml*)	Luteinizing hormone (LH) in the blood. LH is made by your pituitary gland. In women, the pituitary sends out LH during the ovulation part of the menstrual cycle
SHBG (*nM*)	Sex hormone-binding globulin (SHBG) or sex steroid-binding globulin (SSBG) is a glycoprotein that binds to androgens and estrogens
Total Testosterone and Age association	Testosterone test that measures the amount of testosterone in the blood specific to the different age groups
Sperm count (*M/ml*)	Sperm count is generally determined by examining semen under a microscope to see how many sperm appear within squares on a grid pattern
Free Testosterone (*pg/ml*)	Testosterone that is not attached to proteins in the blood
C-reactive protein (*mg/dl*)	A protein made by the liver. CRP levels in the blood increase when there is a condition causing inflammation somewhere in the body
Free androgen index	A free androgen index (FAI) is a ratio figured out after a blood test for testosterone. It’s used to see whether you have abnormal androgen levels
Urinary total testosterone (*ng/mg cr*)	Total amount of testosterone found in urinary that increases chance of conception
Urinary creatinine (*mg/ml*)	A creatinine urine test measures the amount of creatinine in your urine. The test can help your doctor evaluate how well your kidneys are functioning
Sperm morphology	Sperm morphology refers to the size and shape of individual sperm
Prolactin (*μUI/ml*)	Human prolactin is a polypeptide hormone of the anterior pituitary with a molecular mass of about 22,800
IIEF-5	The International Index of Erectile Function – Erectile Function (IIEF-EF) domain score is a patient questionnaire used to measure various aspects of erectile performance and assess disease severity in efficacy trials concerning ED
Menstrual dysfunction	Menstrual dysfunction is common, with approximately 9–30% of reproductive-aged women presenting with menstrual irregularities requiring medical evaluation
PCOS	Polycystic ovary syndrome (PCOS) is a condition that affects a woman’s hormone levels. Women with PCOS produce higher-than-normal amounts of male hormones. This hormone imbalance causes them to skip menstrual periods and makes it harder for them to get pregnant
Hirsutism	Hirsutism (HUR-soot-iz-um) is a condition in women that results in excessive growth of dark or coarse hair in a male-like pattern — face, chest and back
Modified FG-score (mean)	The modified Ferriman-Gallwey (mFG) score grades 9 body areas from 0 (no hair) to 4 (frankly virile), including the upper lip, chin, chest, upper abdomen, lower abdomen, thighs, back, arm, and buttocks
Androstendione (*nmol/l*)	androstendione is an endogenous weak androgen steroid hormone and intermediate in the biosynthesis of estrone and of testosterone from dehydroepiandrosterone
Dehydroepiandrosterone (*nmol/l*)	Dehydroepiandrosterone, also known as androstenolone, is an endogenous steroid hormone.
Estrone (*pmol/l*)	Estrone, also spelled oestrone, is a steroid, a weak estrogen, and a minor female sex hormone. It is one of three major endogenous estrogens, the others being estradiol and estriol
Estronesulfate (*pmol/l*)	Estrone sulfate (E1S) is an estrogen conjugate that serves as a stable circulating reservoir of estrogen, and levels of E1S are the highest among estrogens in postmenopausal women
Follicular phase length	The follicular phase is often the longest part of your menstrual cycle. It’s also the most variable phase
Luteal phase length	The luteal phase is the second phase of your cycle – after ovulation and before your period
AMH (*pmol/l*)	Anti-Müllerian hormone, also known as Müllerian-inhibiting hormone, is a glycoprotein hormone structurally related to inhibin and activin from the transforming growth factor beta superfamily, whose key roles are in growth differentiation and folliculogenesis
DHEAS	Dehydroepiandrosterone, also known as androstenolone, is an endogenous steroid hormone. It is one of the most abundant circulating steroids in humans, in whom it is produced in the adrenal glands, the gonads, and the brain
Amenorrhea	Amenorrhea (uh-men-o-REE-uh) is the absence of menstruation — one or more missed menstrual periods

### Effects of bariatric surgery on fertility in men

#### Sex hormones:

Sex hormones were the most common outcomes assessed and showed significant improvement in all studies with male patients (22–24, 26–28). Overall, weight loss after bariatric surgeries led to an increase in total and free testosterone levels and a reduction in estradiol levels. Facchiano et al. (2013) reported significant improvements for all outcomes (Sex hormones and sex hormone binding globulin), and also found that testosterone improvement was affected by age; men under 35 showed greater increases in both free and total testosterone compared to men over 35 after surgery (27). Three studies compared bariatric surgery to a control or “no surgery” group (24–26) and found significantly greater hormonal improvements in bariatric surgery groups compared to the control groups. Hammoud et al. (2009) compared mean weight changes after two years in a RYGB group and control group and reported significant differences between the two groups for E2, TT, SHGB, FT. Samavat (2018) also reported a significant difference between an operated and non-operated groups for all hormonal outcomes measured.

#### Seminal outcomes:

Among those four studies that examined semen or sperm quality, two studies reported some improvements in seminal parameters. Sperm viability and volume were significantly improved after the surgery compared to the control group in one study (24), and sperm count was significantly improved in a sub-group analysis indicating that patients with azoospermia or oligospermia had significantly increased sperm counts while those patients with normal sperm counts at baseline did not show any difference after the surgery (22). Sperm volume, motility, or morphology did not show any difference in the sub-group analysis. The other two studies did not find any significant improvement in seminal outcomes after the surgery (23, 25).

#### Sexual function and satisfaction:

Hammoud et al. (2009) reported a positive association between bariatric surgery and patients’ sexual quality of life. Legro et al. (2015) did not show any significant improvement in male erectile function, but did show a trend of improvement by 12 months after surgery (p=.13). Reis et al. (2012) used the International Index of Erectile Function (IIEF-5) questionnaire and reported a significant mean score improvement at 24 months after the surgery within the surgery group as well as a significant mean difference compared to the control group (p<.05).

### Effects of bariatric surgery on fertility in women

#### Sex hormones:

Three studies measuring various hormones related to fertility reported a significant decrease of testosterone and an increase of SHBG in women after surgery (29–31). The most significant changes in SHBG occurred within one month after bariatric surgery (30) and lasted until the 12 month follow-up (29, 31). Two studies also reported significant increases in estradiol (29, 30). Other hormones including FSH and LH did not show any significant changes after the surgery. Two studies also reported that hirsutism in women was resolved likely due to improved hormonal balance after the surgery (32, 33).

#### Menstrual cycles:

Six out of 8 studies measuring menstrual cycles in bariatric patients reported significant improvements in regularity and length (30–35). For example, menstruation cycle became regular at 12 months after RYBG in 85% of women with oligo-/amenorrhea (31), the rate of irregular menstruation improved from 60% to 20% after LSG (33), and menstrual dysfunction rate decreased 12.4% after various bariatric surgical procedures (34). Legro et al. (2012) measured multiple menstrual cycle parameters in 9 patients and found mixed results. Obesity is associated with increased length of menstrual cycles, mainly due to lengthening of the follicular phase, so after the bariatric surgery, the study found that the patients’ mean follicular phase length was 6.5 days shorter within 3 months after surgery and 7.9–8.9 days shorter 6–24 months after surgery (p<0.001) and also found a significant decrease in the overall menstrual cycle length at 6 months after surgery (p=0.04) but not at 12 months (30). Goldman et al. (2016) compared the effects of RYGB vs. AGB on menstrual cycle iregularity and found that AGB had greater improvement compared to both no surgery and RYGB groups (OR 0.23 and 0.33, respectively).

#### PCOS:

Two studies measuring PCOS as an out-come reported a significant improvement in 3–12 months (31, 34). Specifically, Kjaer et al. (2017) defined PCOS according to the Rotterdam criteria. Preoperatively, 25% or 8 patients out of 31 had PCOS. After three months only one of the 8 patients still fulfilled PCOS criteria, and after 12 months, none were classified as PCO (31). In the study by Edison et al. (2016), PCOS was evaluated based on the number of patients with PCOS diagnosis before and after bariatric surgery. PCOS was diagnosed in 1,298 patients before surgery and 1,106 patients after surgery which is a 14.8% decrease (p<0.001) (34).

#### Conception/pregnancy:

Jamal et al. (2012) reported a conception rate of 100% for previously infertile PCOS subjects. Four other studies reported improved pregnancy rates after bariatric surgery; seven out of 15 women who were unsuccessful in becoming pregnant became pregnant (46.7%) after LSG (33), five out of nine participants conceived after surgery (30), 83.3% of women who were unable to become pregnant were pregnant after intragastric balloon (36), and 62.7% who could not conceive became pregnant after various surgeries including intragastric balloon, AGB, LSG, and RYGB (37). Musella et al. (2012) also found that BMI and the degree of weight loss after surgery were significant predictors of pregnancy. In contrast, Goldman et al. (2016) and Laurino et al. (2012) reported no significant improvement in conception or pregnancy.

#### Sexual function and satisfaction:

Two studies that examined sexual function, satisfaction, or intimate relationship reported mixed results. One study found a significant improvement in sexual function using the Female Sexual Function Index scale at 12 month follow-up (30), while the other study did not find any significant results in intimate relationship, frequency of sex, or sexual satisfaction (38).

## Discussion

From this systematic review, eighteen studies met the inclusion criteria involving fertility outcomes as a result of bariatric surgery in men and women. Overall, the evidence from this review indicated that fertility parameters including sex hormones in both men and women, seminal outcomes in men, menstrual cycle and PCOS outcomes in women, and sexual function in both men and women improved due to significant weight loss after various bariatric surgeries. Conception or pregnancy was examined only in women and no study with male patients asked whether the participants actually conceived a child with their partners after the bariatric surgery.

Evidence about the effects of bariatric surgery on male fertility.

All studies reported that total testosterone in male patients increased after bariatric surgery. All but one study measured SHBG and found a significant increase in SHGB after bariatric surgery, indicating consistently strong evidence of positive impacts of bariatric surgery on total testosterone and SHBG levels. One article that did not report SHGB measured an increase of prolactin (PRL) levels that enhances luteinizing hormone receptors in Leydig cells which secrete testosterone (39). Four articles reported that estradiol decreased after bariatric surgery, allowing more hormonal balance to be reached in comparison to testosterone (24, 26–28). The same four articles reported that FSH increased after bariatric surgery. Three articles reported an increase in LH, a hormone that binds with Leydig cells to secrete testosterone after bariatric surgery (24, 27, 28).

Unlike sex hormones, evidence of the bariatric surgery effects on seminal outcomes was inconsistent across the studies. Only four studies assessed semen or sperm quality and two of those studies reported some positive outcomes after bariatric surgery. Bardisi et al. (2016) indicated that there might be an interaction between an initial sperm count status and bariatric surgery. Those patients who had azoospermia or oligospermia may benefit from the bariatric surgery.

### Evidence about the effects of bariatric surgery on female fertility:

Articles that assessed fertility in women who underwent bariatric surgery focused on the evaluation of sex hormones, PCOS, menstrual status, hirsutism, pregnancy outcomes. Those articles tended to include women of reproductive age and sometimes included PCOS as an inclusion criterion to determine whether it is improved after bariatric surgery.

Estradiol in women helps with the growth and development of female sex organs, including the uterus (40). Two of the three articles reported estradiol increasing after bariatric surgery. One study reported that estradiol decreased only at 6 months, possibly due to early follow up after bariatric surgery. In females, an acute rise of LH triggers ovulation and development of the corpus luteum. One article reported that the ratio between LH and FSH increased after bariatric surgery to improve fertility (31). As mentioned above, SHBG binds to sex hormones in both men and women and disperses the hormones throughout the body to increase fertility and maintain hormonal balance. Three articles reported that SHBG increased after bariatric surgery (29–31). Hirsutism is a condition of unwanted, male-pattern hair growth in women. Two articles mentioned hirsutism decreasing or being cured after bariatric surgery, conveying hormonal balance being achieved after the surgery (32, 33).

Six articles used menstrual status reaching normal levels, measuring dysfunction, irregularity, and menses length before and after bariatric surgery as indicators of improvement (30–32, 34, 35). Four reported on pregnancy outcomes post bariatric surgery for women (32, 33, 36, 37). Two of those four studies specifically reported pregnancy outcomes increasing after bariatric surgery for women (36, 37). DHEAS improves fertility by increasing the androgen levels within ovary environment to a normal range. Two discussed free androgen index and decreased dehydroepiandrosterone after bariatric surgery (29, 31).

PCOS is a hormonal disorder common among women of reproductive age which causes infrequent or prolonged menstrual periods or an excess of male hormone levels (41). Specifically, androgen is a hormone most commonly known in males for reproductive activity. In females, androgen’s main purpose is to be converted to estrogen. However, excess amounts of androgen can cause females to exhibit masculine characteristics, such as facial hair. The data from research studies in this systematic review conveys a decreasing number of patients qualifying for PCOS diagnosis after bariatric surgery with decreasing levels of androgen in female patients. PCOS may also affect the ovaries by causing them to fail to regularly release eggs (41). The inclusion of PCOS as a measure of fertility is important because of its impact on reproductive factors; however, only two articles assessed PCOS as an outcome and both of these studies reported that the symptoms of PCOS were significantly improved after bariatric surgery (31, 34).

Two articles had no significant information about fertility outcomes among women after bariatric surgery (1, 38).

### Strength of overall evidence:

The strongest evidence from this review was the impact of bariatric surgery on sex hormones in both men and women. The sex hormones that are present in men and women typically flow through the body to allow for fertility and hormone balance. However, in obese patients, sex hormones, such as estrogen and testosterone stored in fatty tissues may result in a hormonal imbalance that causes infertility, PCOS, and irregular menstrual cycles. Once the patients receive bariatric surgery and begin to lose the fat tissues rapidly, all sex hormones stored in those cells are flushed throughout the body, increasing fertility. As a result, bariatric surgery improves fertility by allowing the body to naturally remove fat tissue and release sex hormones to restore fertility and hormonal balance, instead of removing fat through cosmetic surgery. This also allows the patients to achieve hormonal balance as female patients will regain a normal menstrual cycle and healthy amount of estrogen and other hormones throughout the body to lower symptoms of PCOS.

Overall, the articles reviewed provide some consistent data in the amount and type of information for male and female patients to suggest that bariatric surgery improves fertility. Although the articles used different types of bariatric surgeries and recorded different variables, the conclusion amongst 17 articles is that fertility improves in obese patients after bariatric surgery. The purpose of this paper was investigating conception/pregnancy as an outcome for both male and female patients as a direct representation of fertility improvement after bariatric surgery. However, all studies reported specific hormones in improving fertility, such as testosterone, estrogen and SHBG. The results of this review indicate that bariatric surgery significantly improves hormonal balance in males and females after bariatric surgery through measurements reported from hormone levels. There is a lack of studies reporting on whether males father a child after bariatric surgery. Future research and studies are needed to report a specific improvement of fertility on both males and females and pregnancy outcomes. Although the hormones are an effective way to measure fertility, a direct pregnancy outcome will provide more support on fertility improvement.

### Limitations:

Of the 265 articles screened, 18 fit the criteria for reporting fertility outcomes after bariatric surgery in men and women. Since only published studies were included, there is a risk for publication bias, and “grey literature” might have been missed. Due to heterogeneity of bariatric surgery types and dependent variables measured as fertility outcomes across the studies, it was also not feasible to conduct a meta-analysis. Although these limitations remain in the systematic review, to our knowledge, this is the first systematic review to examine and summarize the effects of bariatric surgeries on fertility outcomes in both men and women.

## Conclusion

The results from this systematic review indicate that fertility improves after bariatric surgery for male and female obese patients. Despite the limited number of articles reporting pregnancy outcomes, hormones measured were used to assess fertility improvements after weight loss. However, further research is needed on direct pregnancy outcomes for both men and women after bariatric surgery. A quantitative assessment is needed to address the inability to conceive for obese patients prior to bariatric surgery and the ability to achieve pregnancy outcomes afterward. Further research is also needed in assessing which type of bariatric surgery is most effective at weight loss and fertility improvements for obesity. These methods will ensure future pregnancy outcome and fertility improvement despite obesity and prior failure.
